# Three‐dimensional culture of dental pulp pluripotent‐like stem cells (DPPSCs) enhances *Nanog* expression and provides a serum‐free condition for exosome isolation

**DOI:** 10.1096/fba.2020-00025

**Published:** 2020-06-28

**Authors:** Farid N. Faruqu, Shuai Zhou, Noor Sami, Fatemeh Gheidari, Han Lu, Khuloud T. Al‐Jamal

**Affiliations:** ^1^ Institute of Pharmaceutical Science King’s College London London UK; ^2^ Genomics Centre King’s College London London UK

**Keywords:** cell‐free therapy, pluripotency, regenerative therapy, spheroids, stem cells

## Abstract

Stem cell‐derived exosomes have been identified as novel cell‐free therapeutics for regenerative medicine. Three‐dimensional (3D) culture of stem cells were reported to improve their “stemness” and therapeutic efficacy. This work focused on establishing serum‐free 3D culture of dental pulp pluripotent‐like stem cells (DPPSCs)—a newly characterized pluripotent‐like stem cell for exosome production. DPPSCs were expanded in regular 2D culture in human serum‐supplemented (HS)‐medium and transferred to a micropatterned culture plate for 3D culture in HS‐medium (default) and medium supplemented with KnockOut™ serum replacement (KO‐medium). Bright‐field microscopy observation throughout the culture period (24 days) revealed that DPPSCs in KO‐medium formed spheroids of similar morphology and size to that in HS‐medium. qRT‐PCR analysis showed similar *Oct4A* gene expression in DPPSC spheroids in both HS‐medium and KO‐medium, but *Nanog* expression significantly increased in the latter. Vesicles isolated from DPPSC spheroids in KO‐medium in the first 12 days of culture showed sizes that fall within the exosomal size range by nanoparticle tracking analysis (NTA) and express the canonical exosomal markers. It is concluded that 3D culture of DPPSCs in KO‐medium provided an optimal serum‐free condition for successful isolation of DPPSC‐derived exosomes for subsequent applications in regenerative medicine.

**Abbreviations**
BM: bone marrow
CM: conditioned medium
DPMSCs: dental pulp mesenchymal stem cells
DPPSCs: dental pulp pluripotent‐like stem cells
EBs: embryoid bodies
ECM: extracellular matrix
ED‐HS: exosome‐depleted human serum
ESCs: embryonic stem cells
EVs: extracellular vesicles
GCCP: Good Cell Culture Practice
GMP: Good Manufacturing Practice
hFLK‐1: human fetal liver kinase‐1
hHNF3beta: human hepatocyte nuclear factor 3‐beta
HKG: housekeeping genes
HPRT: hypoxanthine guanine phosphoribosyltransferase
HS: human serum
iPSC: induced pluripotent stem cells
KO: KnockOut™ serum replacement
LIF: leukemia inhibitory factor
MSCs: mesenchymal stem cells
NTA: nanoparticle tracking analysis
Oct4: octamer‐binding transcription factor 4
qRT‐PCR: quantitative reverse transcription polymerase chain reaction
Sox2: sex determining region Y‐box 2
SSEA‐4: stage‐specific embryonic antigen‐4
TSG101: tumor susceptibility gene 101
β2M: beta‐2‐microglobulin

## 1 INTRODUCTION

Following an injury stimulus, various intracellular and intercellular pathways are activated and coordinated in order to restore tissue integrity and homeostasis.^1^, ^2^ The wound repair process involves three main overlapping but distinct phases: inflammation, new tissue formation, and remodeling.^1^, ^2^ In humans, this repair process generally results in fibrotic scar tissues consisting largely of disorganized extracellular matrix (ECM) and few cells (mainly fibroblasts).^2^ Repair resulting in fibrosis is undesirable due to the loss of original tissue function, thus possessing the risk of causing further damages.^2^ Regenerative medicine is defined as the repair, replacement, or regeneration of cells, tissues, or organs to restore impaired function resulting from any cause, including congenital defects, disease, trauma, and aging.^3^ Most often, these approaches harness, stimulate, and support the body's own self‐healing capacity in mediating the regenerative process.^3^

Stem cells serve as the body's endogenous system of regeneration and repair, and thus has attracted interest for use in regenerative medicine.^4^ The use of pluripotent stem cells (ie, cells having the ability to differentiate into the cells from all three embryonic germ layers) such as embryonic stem cells (ESCs) and induced pluripotent stem cells (iPSCs) in regenerative therapy commonly involves subjecting the cells to a directed differentiation into the progenitor cells of the tissue of interest prior to transplantation/engraftment to proliferate and replace damaged tissues.^5^, ^6^, ^7^, ^8^ A class of multipotent stem cells called mesenchymal stem cells (MSCs) represent the most extensively used stem cell in regenerative medicine with around 800 ongoing clinical trials.^9^, ^10^ The attractions for using MSCs in regenerative therapy include their multipotent differentiation potential, unassociated with ethical issues surrounding cell acquisition from their primary sources, and having low immunogenicity following allogeneic transplants.^9^, ^11^, ^12^

A new population of stem cells with pluripotent‐like characteristics was recently isolated from adult tissue and are referred to as dental pulp pluripotent‐like stem cells (DPPSCs).^13^, ^14^ These cells were isolated from the dental pulp of the third molar, and were found to have a similar expression profile to that of embryonic expression pattern (SSEA‐4^+^, OCT4^+^, NANOG^+^, hFLK‐1^+^, hHNF3beta^+^, Nestin^+^, Sox2^+^, Lin28^+^, Myc^+^, CD13^+^, CD105^+^, CD34^‐^, CD45^‐^, CD90^low^, CD29^+^, CD73^low^, STRO‐1^low^, and CD146^‐^).^13^, ^14^ DPPSCs also showed distinct pluripotent characteristics such as teratoma formation upon subcutaneous injection in mice, as well as being able to undergo in vitro differentiation into cells of endoderm (hepatocyte like), mesoderm (osteoblast like), and ectoderm lineages (neuron like).^13^, ^14^ They were shown to have genomic (ie, karyotypic) stability up until 65 passages.^14^ DPPSC is unique where it is the only pluripotent‐like stem cell population to date that originates from adult tissue. DPPSC is a viable alternative pluripotent stem cell source since the third molar is readily accessible as medical waste following wisdom tooth extraction procedures and therefore is not associated with any ethical issues.^13^, ^14^ The dental pulp of the third molar is an adult tissue which develops last in humans. It has been reported that due to its relatively early stage of growth, it provides a high yield of dental pulp tissue for DPPSC isolation.^13^, ^14^, ^15^ The regenerative capability of DPPSC has also been reported where DPPSC promoted wound healing and delayed muscular dystrophy in vivo.^16^

The regenerative effects of stem cells, in particular MSC, depended largely on the paracrine effects of the cells.^17^, ^18^ It was demonstrated that the paracrine regenerative effects were mediated by vesicles referred to as exosomes.^19^, ^20^ Since then, various studies have investigated the therapeutic effect of MSC‐derived exosomes (Exo_MSC_) in various in vitro and in vivo settings.^21^, ^22^, ^23^ Exosomes are a subtype of extracellular vesicles (EVs) secreted by various cell types,^24^, ^25^ as well as being present in various physiological fluids.^26^, ^27^, ^28^ Structurally, exosomes are phospholipid bilayer membrane‐bound vesicles containing an aqueous core and have a hydrodynamic size range ranging from 50 to 200 nm in diameter.^29^, ^30^, ^31^ Exosomes play a role in intercellular communications, where they inherently carry biomolecules such as proteins^32^, ^33^ and nucleic acids,^34^, ^35^ which upon delivery into the recipient cells were processed and elicited functional outcomes. Given their endosomal origin, exosomes are distinguished from other EV subtypes by the enrichment of proteins involved in their biogenesis pathways such as tetraspanins (eg, CD63, CD9, and CD81), Alix, and TSG101.^29^

Culturing cells in a three‐dimensional (3D) format to form spheroid‐like structures was reported to better mimic the in vivo cellular environment, subsequently influencing their behavior and gene expression.^36^, ^37^ For pluripotent stem cells such as ESCs and iPSCs, the spheroid‐like structures formed during the 3D culture prior to their subsequent differentiation are referred to as embryoid bodies (EBs),^38^ and this recapitulates many aspects of cell differentiation into derivatives of all three germ layers by allowing the formation of complex assembly of cell adhesions and intercellular signaling relating to early embryogenesis.^39^, ^40^ Hence, in vitro differentiation mediated by EB formation were reported to exhibit advantages such as faster and more efficient differentiation into various tissue of interest.^41^, ^42^ Recapitulation of the in vivo environment by spheroid formation in 3D culture was also proven to be beneficial to MSC culture, in particular the enhancement of their potential therapeutic efficacy as compared to MSC cultured in 2D such as enhanced anti‐inflammatory effects, angiogenesis, anti‐fibrotic regeneration, and differentiation potential.^43^, ^44^, ^45^, ^46^

Three‐dimensional culture of DPPSCs for purpose other than exosome isolation has been reported.^13^, ^14^, ^47^ Given the favorable 3D culture condition of stem cells as opposed to the conventional 2D monolayer format in terms of in vivo mimicry, signaling networks, and enhanced therapeutic efficacy, it was hypothesized that exosomes derived from 3D culture of stem cells would also exhibit enhanced therapeutic efficacy for use in regenerative medicine. This work therefore aimed to establish a viable serum‐free 3D culture condition for DPPSCs to enable exosome isolation from the stem cells for potential use in regenerative therapy, without adversely affecting the phenotype of the parent cells.

## 2 MATERIALS AND METHODS

### 2.1 Preparation of media for DPPSC culture

#### 2.1.1 Base medium

Sixty percent of Dulbecco's modified Eagle's medium (DMEM)—low glucose (1000 mg/L, Sigma‐Aldrich)—was mixed with 40% MCDB‐201 medium (Sigma‐Aldrich). MCDB‐201 medium was prepared by dissolving 3.51 g of the MCDB‐201 powder in 200 mL deionized water, adjusted to pH 7‐7.5 with NaOH (Fisher Scientific). The DMEM‐MCDB‐201 mixture was then supplemented with 1% penicillin/streptomycin (Thermo Fisher Scientific), 1% GlutaMax^®^ (Thermo Fisher Scientific), 1% SITE (Sigma‐Aldrich), 0.1 mmol/L l‐ascorbic acid 2‐phosphate sesquimagnesium salt hydrate (Sigma‐Aldrich), 0.1 ng/mL murine EGF (PeproTech), 1 ng/mL human recombinant PDGF‐BB (PeproTech), and 1% chemically defined lipid concentrate (Thermo Fisher Scientific).

#### 2.1.2 FBS‐supplemented medium (FBS‐medium)

The base medium for FBS‐medium was prepared as above but with slightly different EGF and PDGF‐BB concentration (10 ng/mL for both), and was additionally supplemented with bovine serum albumin (BSA, 0.2 mg/mL, Sigma‐Aldrich), linoleic acid‐BSA (LA‐BSA, 0.8 mg/mL, Sigma‐Aldrich), and murine leukemia inhibitory factor (LIF, 10^3^ units/mL, Merck). The base medium was then supplemented with 2% FBS (First Link).

#### 2.1.3 Human serum‐supplemented medium (HS‐medium)

DPPSC base medium was supplemented with 1% human serum (Biowest).

#### 2.1.4 Exosome‐depleted FBS‐ (ED‐FBS) and HS‐supplemented medium (ED‐HS)

Both ED‐FBS and ED‐HS were prepared by subjecting unprocessed FBS and HS to ultracentrifugation (Optima™ XPN‐80, Beckman Coulter) at 100 000 g for 18 hour at 4°C and collecting the top two layers of the resulting supernatant. Base medium was then supplemented with 1% ED‐HS to prepare the ED‐HS‐medium (ED‐FBS medium was not used for DPPSC culture).

#### 2.1.5 KnockOut™ serum replacement‐supplemented medium (KO‐medium)

Base medium was supplemented with 20% KnockOut™ serum replacement (Thermo Fisher Scientific), 1% MEM non‐essential amino acids (Sigma‐Aldrich), and 0.1 mmol/L β‐mercaptoethanol (Sigma‐Aldrich).

#### 2.1.6 BIT 9500™ serum substitute‐supplemented medium (BIT‐medium)

Base medium was supplemented with 20% BIT 9500™ serum substitute (STEMCELL Technologies, Grenoble, France).

### 2.2 2D culture of DPPSCs

DPPSCs were provided by Prof. Maher Atari (Universitat Internacional de Catalunya). Cells were isolated from healthy human third molars extracted for orthodontic and prophylactic reasons according to the approval of the Committee on Ethics in Research (CER) of the Universitat Internacional de Catalunya (Spain) under the protocol code BIO‐ELB‐2013‐04 as previously described,^16^ and shipped frozen. For culture of newly thawed cryopreserved cells, a 175 cm^2^ flask (Fisher Scientific) was pre‐coated with fibronectin (100 ng/mL in PBS – 15 mL/flask, Merck) overnight in the incubator (37°C, 5% CO_2_). Thawed cell suspension was added to 15 mL HS‐medium and centrifuged at 500 *g* for 10 minutes at 4°C. The supernatant was discarded and the DPPSCs were resuspended in 1 mL of fresh HS‐medium. The precoated flask was filled with 15 mL (min. volume) HS‐medium after removal of the fibronectin solution, and DPPSC suspension was added to the flask. The medium was replaced the next day, and the cells were monitored and passaged when they reach 40% confluency. For passaging, 3 mL trypsin (Thermo Fisher Scientific) was added to the flask and incubated for 3 minutes in the incubator, and the trypsinization was neutralized with 3 mL HS‐medium. The cell suspension was then centrifuged (500 g, 10 minutes, 4°C), and the DPPSC pellet resuspended in 500 µL fresh HS‐medium. DPPSCs were seeded in new 175 cm^2^ flasks (precoated with fibronectin ≥1 hour prior to seeding) at a density of 100 or 150 cells/cm^2^, of which they will take 4 or 3 days to reach 40% confluency, respectively. The same protocol was followed for 2D culture of DPPSCs in other media, where DPPSCs were washed two times with PBS (Thermo Fisher Scientific, Paisley, UK) after being detached from their old flasks, and added to new flasks (precoated with fibronectin) containing the respective media.

### 2.3 3D culture of DPPSCs

3D culture of DPPSCs were carried out in micropatterned 24‐well culture plates called Aggrewell™ plates (STEMCELL Technologies). The Aggrewell™ plate was prepared by first adding 500 µL anti‐adherence rinsing solution (STEMCELL Technologies) to each well, and the plate was centrifuged at 2000 *g* for 2 minutes to remove bubbles. The plate was then incubated at room temperature (RT) for 30 minutes to 2 hours. In the meantime, DPPSCs were harvested from 2D culture, washed twice with PBS, resuspended in base medium, and kept on ice. After incubation, the rinsing solution in the Aggrewell™ plate was discarded and each well was washed with 500 µL PBS. HS‐medium (500 µL) was added to each well, and the plate was centrifuged again at 2000 *g* for 2 minutes. The medium was discarded, and 800 µL to 1 mL fresh HS‐medium containing DPPSC at a density of 1.2 × 10^5^ cells/well (ie, 100 cells/microwell) were added to each well of the Aggrewell™ plate. For 3D culture of DPPSCs in other medium supplementation, the wells of the Aggrewell™ plate was washed with the corresponding medium instead, and the cells were added to the medium at the same density before seeding. The cells in each plate were mixed thoroughly by pipetting to ensure even distribution of the cells in each microwell. The plate was then centrifuged again at 500 *g* for 5 minutes to collect the cells at the bottom of the microwells, and this was checked by observation under the microscope (CKX41, Olympus) at 10× magnification. The cells were kept in the incubator and left undisturbed for at least 3 days. The medium was then changed after 3 days (with very gentle aspiration and dispensing of media as the cells/spheroids are not adherent), and the cells were imaged under the microscope at 10× and 40× magnification (MicroPublisher 3.3 RTV, Teledyne QImaging). The medium was then changed every 2‐3 days and the culture maintained for 24 days. All conditioned medium collected during medium changing were stored at 4°C for exosome isolation.

To harvest the DPPSC spheroids, base medium was added to the wells of the Aggrewell™ plate (~1 mL per 6 wells) and pipetted up and down several times thoroughly to resuspend the spheroids. Spheroids are handled using 1‐ml pipette with its tip cut to provide a bigger orifice and avoid spheroid disintegration.

### 2.4 Culture of umbilical cord‐derived mesenchymal stem cells (ucMSCs)

Umbilical cord‐derived mesenchymal stem cells were provided by Prof. Francesco Dazzi (Comprehensive Cancer Centre, King's College London). ucMSC culture was done in 175 cm^2^ flasks without requiring any precoating. When thawing cells, the medium used was MEM‐α (Thermo Fisher Scientific) supplemented with 10% FBS (First Link), 1% penicillin/streptomycin (Thermo Fisher Scientific), and 1% GlutaMax^®^ (Thermo Fisher Scientific). On the following day, old medium was discarded and replaced with medium supplemented with 5% human platelet lysate (STEMCELL Technologies) instead. ucMSCs were allowed to grow until they reach 80%‐90% confluency before passaging. Passaging the ucMSCs follows conventional trypsinization protocol, and seeding was done following conventional ratio‐based protocol (eg, 1:3 from original flask) without requiring a particular seeding density in the platelet lysate‐supplemented medium (default medium).

### 2.5 Protein quantification by absorbance at 280 nm

This was carried out using NanoDrop™ 1000 spectrophotometer (Thermo Fisher Scientific). The pedestal was cleaned with absolute ethanol and water prior to carrying out measurements. Sample of interest (1.5 μL) was added to the pedestal, and the absorbance at 280 nm was read in triplicates and the results were expressed as mean ± SD. The pedestal was cleaned with water in between sample measurement to avoid false measurements.

### 2.6 Spheroid size analysis

Size of the resulting spheroids was measured and analyzed using the ImageJ software (NIH). Measurements of the spheroids were done on the images captured by bright‐field microscopy at 10X magnification while still in the Aggrewell™ plates and when in suspension after harvest. Results were expressed as mean ± SD, where n = 15‐30.

### 2.7 Quantitative RT‐PCR (qRT‐PCR) for pluripotency gene expression analysis

#### 2.7.1 RNA extraction

RNA extraction was carried out using TRIzol™ (Thermo Fisher Scientific) with slight modifications from the manufacturer's instructions. Briefly, cells/spheroids were washed twice with 1 mL PBS after harvest. TRIzol™ (400 µL) was added to the cells and the solution was pipetted up and down vigorously several times to homogenize the cells. The solution was then heated at 55°C for 10 minutes and later vortexed for 5 seconds (done twice to ensure maximum cell lysis). The cell lysate in TRIzol™ were transferred to Phasemaker™ tubes (Thermo Fisher Scientific), and 80 µL chloroform (Fisher Scientific) was added to the solution (200 µL chloroform per 1 mL TRIzol™ used). The mixture was vortexed for 30 seconds, and allowed to incubate at RT for 20 minutes. The mixture was then centrifuged at 15 000 *g* for 15 minutes at 4°C. After centrifugation, the clear aqueous phase (RNA‐containing fraction) was aspirated out and transferred to an RNase‐free tube. Isopropanol (200 µL, Fisher Scientific) was added to this aqueous phase (500 µL chloroform per 1 mL TRIzol™ used), and the mixture was inverted up and down for ~30 times and left to incubate at RT for 10 minutes. This mixture was then centrifuged at 15 000 *g* for 15 minutes at 4°C. The supernatant was carefully pipetted out and the pellet was resuspended in 75% ethanol (Sigma‐Aldrich). The solution was then vortexed for 10 seconds and centrifuged at 7500 *g* for 5 minutes at 4°C. The supernatant was carefully pipetted out and the pellet was left to air‐dry. Once dry, the pellet was resuspended in 35 µL of TE buffer (Sigma‐Aldrich). RNA concentrations were measured using NanoDrop™, alongside the quality (ie, the A_260nm_/A_280nm_ ratio). Only RNA samples with A_260nm_/A_280nm_ ratio ≥1.5 were used for cDNA synthesis and PCR/qRT‐PCR experiments. RNA stocks were stored at −80°C until use.

#### 2.7.2 cDNA preparation (reverse transcription)

cDNA synthesis was performed using the QuantiTect^®^ Reverse Transcription kit (Qiagen), following the instructions provided by the supplier. Equal amounts of RNA (160 ng) from each cell clone were used for the cDNA synthesis. It was assumed that the amount of cDNA produced at the end of the reaction is equal to the amount of RNA used for the reaction. The reverse transcription products were then made up with nuclease‐free water to obtain 5 ng/µL cDNA stocks and stored at −20°C until use.

#### 2.7.3 Primer design and sequences

Primers for all housekeeping and target genes were designed using the Primer‐BLAST online tool (NIH, USA – https://www.ncbi.nlm.nih.gov/tools/primer‐blast/), and the exon template sequence for each gene were obtained from the NCBI Gene database (NIH, USA – https://www.ncbi.nlm.nih.gov/gene). Primers were designed using the following criteria: (a) Primers must span an exon‐exon junction, (b) Range of target PCR product falls within 7‐250 base pairs (bp), (c) Range of primer melting temperatures (T_m_) fall within 60 ± 3°C, (d) Minimum of seven and five nucleotides present at 5′ and 3′ end of exon, respectively, (e) At least one of the last two nucleotides at the 3′ end of the designed primer ends with C/G, (f) The ΔG value for the homodimer (of each primer of the pair) and heterodimer tendency of the primers are > −6 kcal/mol, and (g) The G/C content of each primer is ≤60%. Thermodynamic parameters of the primers were analyzed using the OligoAnalyzer online tool (IDT – https://www.idtdna.com/calc/analyzer), with “qPCR” chosen for the “Parameters set” option. Sequences of primers used are as follows: *GAPDH*, forward, 5′‑TGTTCCAATATGATTCCACCCA‑3′, reverse 5′‑CAAATGAGCCCCAGCCTTC‑3′; *18S rRNA*, forward, 5′‑GTTGAACCCCATTCGTGATG‑3′, reverse, 5′‑CCATCCAATCGGTAGTAGCG‑3′; *Oct4A*, forward, 5′‑CTTCGCAAGCCCTCATTTCACC‑3′, reverse, 5′‑CCAGGTCCGAGGATCAACC‑3′; *Nanog*, forward, 5′‑CTGATTCTTCCACCAGTCCC‑3′, reverse, 5′‑AGGTCTTCACCTGTTTGTAG‑3′. All primers were purchased from IDT (Leuven, Belgium), reconstituted in TE buffer to obtain 10 µmol/L stocks upon arrival and stored at −20°C until use.

#### 2.7.4 Relative expression analysis of pluripotency genes

The qRT‐PCR reaction mixture was prepared to contain 1X SsoAdvanced™ SYBR^®^ Green Supermix (Bio‐Rad), 500 nmol/L forward and reverse primers, 10 ng cDNA and made up to 20 µL total reaction volume with nuclease‐free water. All components were kept on ice after thawing, and the reaction mix preparation was done in a 96‐well PCR plate placed on cold blocks (three replicates per condition). The PCR plate was sealed with a clear adhesive film and centrifuged briefly to ensure all liquid was collected at the bottom of each well. The plate was then placed in the ABI 7500 Real‐Time PCR System (Thermo Fisher Scientific), and the reaction was run according to the cycling conditions: initial denaturation—95°C, 60 seconds; amplification (40 cycles)—95°C, 15 seconds and 65°C (annealing temperature), 60 seconds; melt curve analysis—instrument default settings. Ct values of each of the housekeeping and target genes were obtained using the 7500 software v2.3 (Thermo Fisher Scientific). The relative expression levels of each target gene in all the cDNA tested were calculated using the 2^−ΔΔCt^ method, using Ct values from hiPSC as the reference.

### 2.8 Exosome isolation from conditioned medium (CM)

This was carried out following a previously published protocol,^48^ with slight modifications. Briefly, CM was filtered through 0.22 µm filter (Merck) and added to polycarbonate ultracentrifuge tubes (Beckman Coulter). Meanwhile, the sucrose solution for the density cushion was prepared as follows: 25% w/w in deuterium oxide (D_2_O). This sucrose cushion (3 mL) was then carefully added to the bottom of the CM in the ultracentrifuge tubes by pipetting 1 mL of the solution at time through a glass pipette. The ultracentrifuge tubes were then placed in a swing‐out rotor (SW45 Ti, Beckman Coulter) and subjected to ultracentrifugation at 100 000 *g* for 1.5 h  at 4°C. After ultracentrifugation, 2 mL was withdrawn and added to 20 mL‐filtered PBS in polycarbonate ultracentrifuge bottles (Beckman Coulter). This was subjected to another round of ultracentrifugation in a fixed‐angle rotor (Type 70 Ti, Beckman Coulter) at 100 000 *g* for 1.5 h  at 4°C. The pellet obtained (ie, the exosomes) was resuspended in 400 µL‐filtered PBS. Exosomes were kept at 4°C and −80°C for short‐ and long‐term storage, respectively.

### 2.9 Nanoparticle tracking analysis

Exosome hydrodynamic size and number were measured by nanoparticle tracking analysis (NTA) using NanoSight LM10 (Malvern Instruments). The exosome sample was first diluted in filtered PBS in a total volume of 0.75‐1 mL to obtain 20‐80 particles in the viewing frame. Samples were injected into the sample chamber of the NanoSight LM10 using a 1‐mL syringe. The modal size and particle count were measured in triplicates, with 30 seconds as the duration for each recording. The temperature for each recording was noted in the software. Results were analyzed using the NanoSight NTA 3.2 software (Malvern Instruments) and were expressed as mean ± standard deviation (SD).

### 2.10 Detection of surface exosomal markers by dot blot

Exosomes (≥1 × 10^10^ p/mL stock concentration) were spotted on a nitrocellulose membrane (Bio‐Rad) (40 µL total—10 µL at a time, dried under a nitrogen stream before addition of the next 10 µL on the same spot). The membrane was then placed in a 50‐mL centrifuge tube with the exosome‐spotted side facing inwards. The tube was filled with 5 mL 3% milk (Sigma‐Aldrich) prepared in TBS‐T. TBS‐T was prepared by dissolving 2.42 g Tris base (Formedium) and 8 g NaCl (VWR Chemicals) in 1 L deionized water, adjusted to pH 7.6 with 0.1% Tween‐20 (Sigma‐Aldrich) added. The membrane was allowed to incubate with the 3% milk for blocking 1 hour at RT on a roller, with the speed set on “Very low.” After blocking, the milk is discarded, and fresh 5 mL 3% milk in TBS‐T (hereon referred to as blocking buffer) was added to the tube. Primary antibodies (CD9 – mouse anti‐human, BioLegend; CD63 and Alix – rabbit anti‐human, Abcam; TSG101 and CANX – rabbit anti‐human, ProteinTech) were added to the blocking buffer (1:1000, one antibody for each tube), and left to incubate on a roller overnight at 4°C. The blocking buffer containing the primary antibodies was discarded, and the membrane was washed three times with 5 mL TBS‐T on a roller (5 minutes at RT for each wash). Fresh blocking buffer (5 mL) was added to the tube after the final wash, and HRP‐conjugated secondary antibody was added to the blocking buffer (goat anti‐mouse – 1:20 000; goat anti‐rabbit – 1:1000; both from Cell Signalling Technology), and was left to incubate on the roller for 1 hour at RT. Blocking buffer containing the primary antibodies were discarded, and the membrane was washed three times with 5 mL TBS‐T as above. After the final wash, ECL substrate (~1 mL per membrane, prepared by 1:1 mix of the two components, Bio‐Rad) was added to the membrane, and left to stand for 3 minutes, protected from light. The membrane was then imaged using the Gel Doc™ system (Bio‐Rad) under 15 minutes exposure time. In case where the signal is too intense using 15 minutes exposure time, the “Faint Bands” or “Intense Bands” were used accordingly. The image obtained was analyzed using the Image Lab™ software (Bio‐Rad).

## 3 RESULTS

### 3.1 Different serums and serum‐free supplementations have variable protein amount

Two different serums were used in this study for DPPSC culture, namely FBS and human serum (HS). The exosome‐depleted versions of both serums (ED‐FBS and ED‐HS) were also prepared for subsequent culture for exosome isolation purpose. Since preparations of exosome‐depleted FBS in cell culture were reported to have reduced growth capacity,^49^ two other serum‐free alternatives were also explored—KnockOut™ serum replacement (KO) and BIT 9500™ serum substitute (BIT). The protein amount of the different serum and serum‐free supplementations was first quantified to have an idea of their differences. FBS and HS recorded the highest protein amount (79.5 and 72.1 mg/mL respectively) (Table 1). These were slightly higher than that of KO (67.6 mg/mL) and was followed by BIT (39.9 mg/mL) and ED‐HS (37.9 mg/mL). ED‐FBS recorded the lowest protein amount with 25.0 mg/mL.

**TABLE 1:** Protein amount in different sera and serum‐free supplementations

Supplementation	Protein amount (mg/mL)
FBS	79.5 ± 2.12
HS	72.1 ± 0.20
ED‐FBS	25.0 ± 0.13
ED‐HS	37.9 ± 0.32
KO	67.6 ± 0.46
BIT	39.9 ± 0.34

Note

Measured using NanoDrop™.

Values are expressed as mean ± SD (n = 3).

### 3.2 Human serum (HS)‐supplemented medium allows expansion of DPPSCs in 2D culture

**FIGURE 1:**
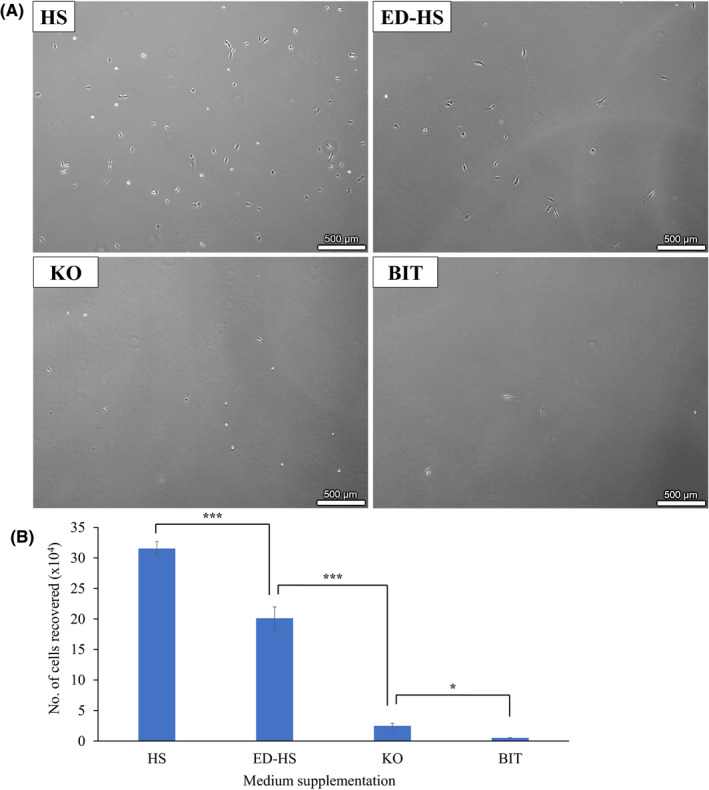
2D culture of dental pulp pluripotent‐like stem cells (DPPSCs) in differentially supplemented exosome‐free media. A, Representative morphology (from three different clones) of DPPSCs cultured in four differentially supplemented media: human serum (HS), exosome‐depleted human serum (ED‐HS), KnockOut™ serum replacement (KO), and BIT 9500 serum substitute (BIT). Cells were seeded at a density of 100 cells/cm^2^ in 175 cm^2^ culture flasks. Images were obtained using bright‐field microscopy under 4× magnification on day 4 of culture. B, Proliferation of DPPSCs in the differentially supplemented media assessed by the number of cells recovered after 4 days. Values are expressed as mean ± SD, where n = 3 different DPPSC clones. One‐way ANOVA was used for statistical analysis (*P < .05, ***P < .001)

DPPSCs were initially expanded in conventional monolayer format (2D culture). In our hands, culture of DPPSC in FBS‐medium was not viable as previously reported (Figure S1; Table S1).^13^, ^14^, ^15^ When switched to HS supplementation, DPPSCs showed the expected morphology across multiple passages (Figure 1A; Figure S2A), and excellent proliferation (Figure S2B; Table S2). In preparation for exosome isolation, DPPSCs were then cultured in medium supplemented with ED‐HS, KO, and BIT. DPPSCs in all culture media showed the expected morphology, but only DPPSCs in HS‐medium reached 40% confluency after 4 days (maximum confluency allowed to avoid spontaneous differentiation) (Figure 1A). DPPSCs in ED‐HS‐medium only reached ~20% confluency within the same time interval, and were not viable in the subsequent passages (data not shown). DPPSCs in KO‐ and BIT‐medium both showed minimal viability on day 4. Correspondingly, DPPSCs in HS‐medium recorded the highest number of cells recovered after 4 days (31.5 × 10^4^ cells), followed by those in ED‐HS‐medium, KO‐medium, and BIT‐medium in descending order (~21.8 × 10^4^, 2.9 × 10^4^, and 0.6 × 10^4^ cells, respectively) (Figure 1B). HS‐medium was therefore chosen as the default medium for 2D expansion of DPPSC.

### 3.3 KO‐medium enables similar DPPSC spheroid formation to that in HS‐medium

**FIGURE 2:**
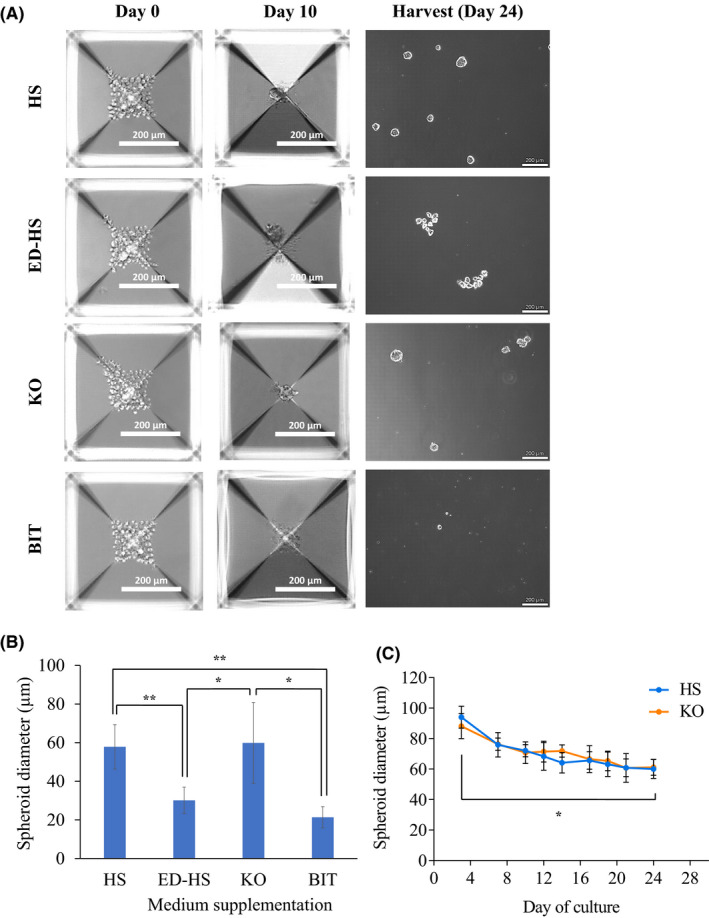
3D culture of dental pulp pluripotent‐like stem cells (DPPSCs) in differentially supplemented medium. DPPSCs were first cultured and expanded in HS‐medium in 2D format to obtain sufficient cells. DPPSCs were seeded at a density of 1.2 × 10^5^ cells/well (100 cells/microwell) of the Aggrewell™ plate for each medium (6 wells/medium). A, Images show the representative morphology of the DPPSC cells or spheroids on day 10, 20, and 24 of culture, where the spheroids/cells are harvested. Images were obtained by bright‐field microscopy under 10× magnification. For day 10 and 20, images are from a single microwell of the Aggrewell™ plate. B, Size analysis of DPPSC spheroids formed in the differentially supplemented media using ImageJ (NIH) software after harvest from the Aggrewell™ plate. C, Size analysis of DPPSC spheroids in HS‐ and KO‐medium throughout the culture period, on the images taken by bright‐field microscopy on each day the medium was changed. Values are expressed as mean ± SD (n = 15‐30 for (B) and (C). One‐way ANOVA was used for statistical analysis in (B) and (C) (*P < .05, **P < .01)

3D culture of DPPSC was carried out in micropatterned 24‐well culture plates called Aggrewell™ 400 plates. Each well in this micropatterned plate comprises of 1200 microwells which take the shape of an inverted pyramid.^50^ Centrifugal force allows the collection of cells at the bottom of the microwell without adherence thereby allowing for the formation of spheroids and hence culturing of the cells in a 3D manner. DPPSCs were initially expanded (in 2D) in the default HS‐medium to obtain sufficient cells prior to the 3D culture. The morphology of the resulting spheroids was observed using bright‐field microscopy throughout the culture period (24 days). DPPSCs formed spheroids on : 6/14/2020, 01:59:54 PM" timestamp="1592123394825">day 10 in HS‐, ED‐HS‐, and KO‐medium, but not in BIT‐medium (Figure 2A). Upon harvesting on day 24, DPPSC spheroids in HS‐ and KO‐medium were of similar size (~60 µm), and both were significantly larger than DPPSC spheroids in ED‐HS‐medium (~30 µm) (Figure 2B). DPPSC in BIT‐medium was found to eventually form spheroids, which were the smallest (~21 µm) and appeared more disintegrated with individual cells observed in suspension (Figure 2A). Given the potential of KO‐medium in enabling the formation of comparably viable spheroids, 3D culture of DPPSC in both HS‐ and KO‐medium was repeated, and it was observed that in both media, DPPSC spheroids started to form as early as day 3 (Figure 2C; Figures [Link], [Link] and [Link], [Link]). It was also noted that DPPSC spheroids in both media showed reduction in size over time, from ~90 µm on day 3, to ~60 µm on day 24 (Figure 2C). Representative images of DPPSC spheroids in both HS‐ and KO‐medium throughout the culture period are shown in Figures [Link], [Link] and [Link], [Link], respectively. In summary, KO‐medium enabled DPPSC spheroid formation of similar viability and morphology as that in the default HS‐medium.

### 3.4 3D culture of DPPSC in KO‐medium improves *Nanog* expression while maintaining *Oct4A* expression

**FIGURE 3:**
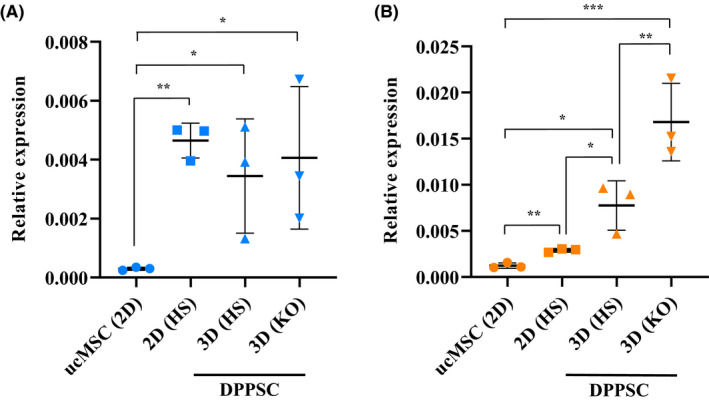
Relative expression levels of pluripotency genes in dental pulp pluripotent‐like stem cells (DPPSCs) cultured in different conditions. cDNA samples were prepared by reverse transcription following RNA extraction from the following cells: human induced pluripotent stem cells (hiPSCs), umbilical cord‐derived mesenchymal stem cells (ucMSCs), 2D DPPSCs in HS‐medium, 3D DPPSCs in HS‐medium, and 3D DPPSCs in KO‐medium. Equal amounts of cDNA samples from each cells were then used in a qPCR to obtain the Ct values of the housekeeping genes (GAPDH and 18S rRNA) and target genes (*Oct4A* and *Nanog*). Using the Ct values of the three genes in iPSC as the reference, the relative expression levels of (A) *Oct4A* and (B) *Nanog* gene in ucMSC and DPPSC (2D and 3D) were calculated using the 2^−ΔΔCt^ method. Each individual sample for each culture condition represents one DPPSC/ucMSC clone. RNA for each sample was extracted from ~14 000 spheroids. One‐way ANOVA was used for statistical analysis (*P < .05, **P < .01, ***P < .001)

Next, the pluripotency‐like characteristics of DPPSC spheroids formed in both HS‐ and KO‐medium were assessed. qRT‐PCR analysis was carried out on the RNA extracted from DPPSC spheroids in both media to determine the expression levels of pluripotency genes *Oct4A* and *Nanog*. DPPSC in 2D culture showed significantly higher *Oct4A* expression compared to that of ucMSCs—a multipotent stem cell (Figure 3A). This is expected as the former is reported to be a pluripotent cell type. *Oct4A* expression of DPPSC spheroids in KO‐medium was similar to that in HS‐medium, of which both showed similar levels to that of DPPSC cells in 2D culture. DPPSC in 2D culture showed the expected higher expression of *Nanog* than that of ucMSC (Figure 3B). *Nanog* expression was also found to be significantly higher in DPPSC spheroids in HS‐medium than that of their 2D counterparts. Interestingly, DPPSC spheroids in KO‐medium showed a significantly higher *Nanog* expression than that in HS‐medium. In summary, the results suggest that 3D culture of DPPSCs in KO‐medium maintained their *Oct4A* expression while increasing the expression of *Nanog* more significantly than that in the default HS‐medium. Combined with spheroid morphology and size results earlier, KO‐medium was considered the optimal medium for 3D culture of DPPSCs for subsequent exosome isolation.

### 3.5 KO‐medium enables exosome isolation from DPPSC spheroids

Having demonstrated that DPPSCs benefited from 3D culture of in KO‐medium in terms of increased *Nanog* expression while maintaining *Oct4A* expression as well as spheroid size and morphology, CM from DPPSC spheroids cultured in KO‐medium were subjected to an exosome isolation attempt. The CM was pooled into two separate samples for the isolation—day 1‐12 and day 13‐24 of culture. Equal volume of unconditioned KO‐medium was also subjected to the same exosome isolation protocol to determine the background level of vesicles/particles, if any.

#### 3.5.1 Physicochemical characterization

**FIGURE 4:**
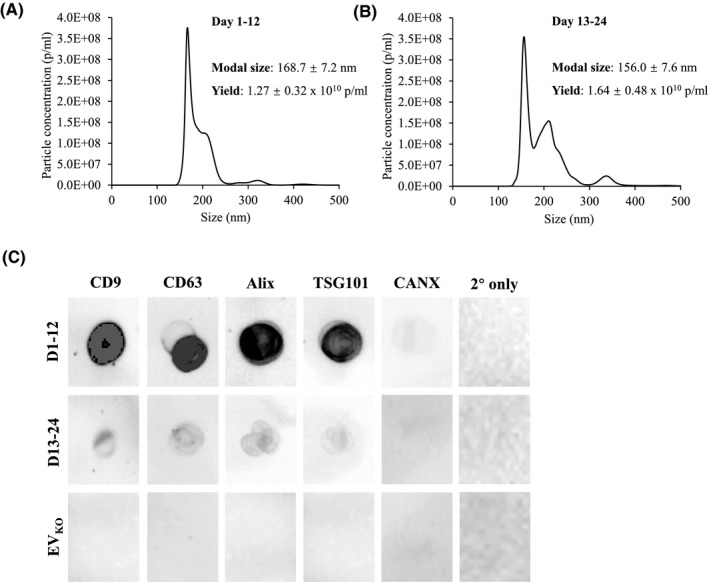
Characterization of EVs isolated from 3D culture of dental pulp pluripotent‐like stem cell (DPPSC) in KO‐medium (EV_DPPSC_). (A, B) Histograms showing results from NTA analysis of EVs isolated from conditioned media (CM) collected from DPPSC spheroids from day 1‐12 and day 13‐24, respectively. The final resultant pellet from the isolation was resuspended in 400 µl PBS. C, Detection of exosomal surface markers CD9 and CD63, luminal markers Alix and TSG101, as well as endoplasmic reticulum‐associated protein Calnexin (CANX) on DPPSC‐derived EV (EV_DPPSC_) by dot blot. Equal number of EV_DPPSC_ (40 µL from 1 × 10^10^ p/mL stock was spotted on the nitrocellulose membrane prior to staining. The same markers were also analyzed on equal number of “EV” derived from unconditioned KO‐medium subjected to the same exosome isolation protocol as the conditioned media above (EV_KO_ – 80 µL from 0.5 × 10^10^ p/mL stock). Samples were also stained with secondary antibodies only as control for nonspecific background signals

The isolated EVs from DPPSCs (EV_DPPSC_) from both culture durations were subjected to NTA for yield and particle size analysis. Both day 1‐12 and day 13‐24 samples recorded similar EV_DPPSC_ yields (~1.3 and ~ 1.6 x 10^10^ p/ml, respectively) (Figure 4A,B). EV_DPPSC_ from both day 1‐12 and day 13‐24 samples also recorded similar particle sizes (~169 nm and ~156 nm, respectively).

#### 3.5.2 Biochemical characterization

The expression of exosomal markers on the isolated EV_DPPSC_ from both day 1‐12 and day 13‐24 were carried out using dot blot using equal numbers of particles from both samples. EV_DPPSC_ from both day 1‐12 and day 13‐24 samples were positive for exosome‐associated tetraspanins CD9 and CD63, as well as the endosome‐associated proteins Alix and TSG101, confirming their endosomal origin during biogenesis (Figure 4C). EV_DPPSC_ from both durations were also found to be negative for the endoplasmic reticulum‐associated protein calnexin (CANX), suggesting minimal contamination from nonexosomal vesicles. Interestingly, a significantly lower signal intensity was detected for all the exosomal markers on EV_DPPSC_ from the day 13‐24 samples compared to that in the day 1‐12 samples. Equal numbers of “EV” isolated from unconditioned KO‐medium (EV_KO_) were also subjected to the same dot blot analysis, and were found to be negative for all the markers tested, which confirmed that the signals detected in the EV_DPPSC_ samples were indeed from the actual isolated EVs and not from background vesicles/particles in the KO‐medium. In summary, the physicochemical and biochemical characterization results provided substantial evidence that 3D culture of DPPSC in KO‐medium has enabled the successful isolation of DPPSC‐derived exosomes (Exo_DPPSC_), and that this should be done for the CM collected from day 1‐12 of culture.

## 4 DISCUSSION

The move away from using FBS in culture of stem cells is not just limited to the phenotypic changes they may cause in the stem cells cultured. Clinical use of stem cells for regenerative therapy relies heavily on their in vitro expansion due to the insufficient amount obtained from primary sources alone.^51^ One major concern is the contamination of FBS‐derived substances in the expanded stem cells for therapy use potentially raising harmful xenogeneic immune responses, either by internalization and subsequent antigen presentation, or by attachment to cell surface and therefore becomes antigenic substrates following transplantation.^52^, ^53^ Such instances of immune responses in other forms of cell‐based therapy have been reported, where patients administered with lymphocytes expanded in FBS‐medium developed arthus‐like reaction.^54^ In another instance, patients administered with autologous T cells expanded in FBS‐medium developed IgG immunity against FBS lipoproteins.^55^ Other concerns of FBS contamination in administering cells for therapy use include the risks of viral and prion and transmission.^56^, ^57^ Contamination of the xenogeneic sialic acid Neu5Gc in culture of stem cells in FBS‐medium of which some were able to be metabolically incorporated into stem cells cultured in FBS have also been reported.^53^ This poses the risk of zoonotic infections of viruses with tropism for the Neu5Gc metabolically incorporated into the proteoglycans of the stem cells, or rendering the stem cells inactive if administered into patients with anti‐Neu5Gc antibodies.^58^, ^59^ The risk of disease transmission from FBS in culture largely stems from FBS not being subjected to stringent screening procedures as routinely done on human plasma/serum after being obtained from donors.^60^ The risks and ethical issues have prompted the use of serum‐free defined medium toward achieving clinical quality cell‐derived samples, compliant to the proposed Good Cell Culture Practice (GCCP) and Good Manufacturing Practice (GMP).^51^, ^61^

A few studies reported the use of HS in cell culture studies and have shown comparable or improved phenotypic outcomes as to using FBS. A study reported that use of HS in culture of human dental pulp stem cells (hDPSC) resulted in their comparable expansion, and later myogenic and osteogenic differentiation, as well as more efficient adipogenic differentiation compared to that done in FBS‐medium.^62^ Another study reported comparable expansion of human bone marrow‐derived MSC (hBM‐MSC) and later their osteogenic and adipogenic differentiation in HS‐medium compared to that in FBS.^63^ Culture of cervical cancer cell lines (HeLa and SiHA) in HS‐medium was reported to result in similar proliferation and migration rate, but higher invasion rate as well as better spheroid formation and integrity when compared to those cultured in FBS‐medium.^64^ The discrepancy in the results obtained from studies comparing HS and FBS for use in cell culture is expected, as the composition and concentration of the different proteins, lipids, and soluble factors will vary in the different species,^56^ and so it might be worth noting that the use of HS for the culture of human‐derived cells in vitro is more likely to better reflect their native in vivo phenotype since HS would contain the more representative factors that human‐derived cells are exposed to in vivo.^65^

In this study, 2D culture of DPPSCs in the differentially supplemented media resulted in only the cells cultured in ED‐HS‐medium showing almost comparable viability to those in HS‐medium, while the cells cultured in KO‐ and BIT‐medium showed minimal viability (Figure 1). There have been reports on using the KO supplement in successful 2D culture of embryonic stem cells (ESC),^66^, ^67^ as well as MSC.^68^, ^69^ This work however is the first to report on the attempt of 2D DPPSC culture using KO‐medium. The composition of the KO supplement was reported, although not entirely, to contain amino acids, vitamins, albumin substitute, insulin substitute, a transferrin substitute, collagen precursors, antioxidants, and trace elements among others, but lacks soluble molecules and factors unattached to albumin, which are present in FBS.^70^ Given that the protein amount in KO and HS are similar (Table 1), lack of these factors are likely to contribute toward the significantly lower viability of DPPSC when cultured in KO‐medium in 2D. Studies that successfully carried out 2D culture of the ESC adopted a feeder‐dependent condition, which most likely contributed toward the secretion of the growth factors lacking in the KO‐medium alone. These studies that used KO‐medium in culturing pluripotent cells were mostly using ESCs or iPSCs, and so the different viability observed with 2D culture of DPPSC in this work could also be a lineage‐related factor.

Interestingly, unlike in 2D culture, DPPSCs were able to form spheroids in 3D culture with comparable size and integrity in KO‐medium as those in HS‐medium. Although a lot of studies on 3D culture of stem cells refers to the 3D structure formed as EBs, they are referred to as spheroids in this work as the term EB is more accurately used when the spheroids were allowed to undergo a differentiation protocol,^38^ while in this work they were not subjected to any differentiation but rather maintained in their undifferentiated state. It has been reported that cells in 3D culture have more activated signaling pathways due to the cells being in contact with each other, which resulted in the release of various growth factors secreted in a paracrine manner.^71^, ^72^ This probably compensated for the lack of certain factors in the KO‐medium and allowed for the spheroids to form in KO‐medium. There were indeed reports on successful EB formation by pluripotent stem cells in KO‐medium.^73^, ^74^ These studies maintained the EB culture for a range of 2‐8 days, and the progression of the EB size and morphology was not analyzed. In this work, it was observed that DPPSC spheroids in both HS‐ and KO‐medium decrease in size with culture duration but still maintaining their integrity (Figure 2C). This is the first of such report and so comparison to other studies were not able to be drawn. Although the scope of this work is to maintain the DPPSCs in an undifferentiated state while still being able to isolate exosomes, it is speculated that DPPSC spheroids in KO‐medium are able to be subjected to directed differentiation, as various stem cells cultured in both 2D and 3D formats were successfully differentiated into several lineages when cultured in KO‐supplemented media.^66^, ^68^, ^69^, ^74^ In contrast, a study reported that the EB formation from ESC in BIT‐medium was not as viable as those in KO‐medium,^75^ which supported the observation on the inability of DPPSCs to form spheroids in BIT‐medium reported in this work.

qRT‐PCR analysis in this work showed that *Oct4A* expression level in DPPSC spheroids in KO‐medium was similar to that in HS‐medium. It is important to note that the *Oct4* gene results in four different transcriptional isoforms, and that only the *Oct4A* mRNA (variant 1) results in the translation of the nuclei‐residing transcription factor implicated in maintaining pluripotency in ESC.^76^ *Oct4* gene was also reported to have six pseudogenes, three of which encodes for proteins highly homologous to the Oct4A protein.^77^, ^78^ *Oct4A* expression analyzed in this work was done using primer sequences validated to be specific for the correct *Oct4A* mRNA and not the pseudogenes.^16^, ^76^ It was demonstrated in this study that DPPSC spheroids in KO‐medium showed a significantly higher *Nanog* expression than their counterparts in HS‐medium (Figure 3B). Oct4 is well known as one of the key factors determining the pluripotency of a cell, and was demonstrated to comprise the quartet of essential transcription factors (Oct4, Sox2, C‐Myc, and Klf4)—the Yamanaka factors—to be expressed in reprogramming somatic cells to a pluripotent state.^79^, ^80^, ^81^ Absence of Oct4 overexpression did not give rise to ES‐like colonies,^79^, ^82^ and it has even been demonstrated that nuclear reprogramming to pluripotency is achievable using only two factors with the aid of a histone deacetylase inhibitor, of which Oct4 was one of them and was indispensable.^83^ These reports undoubtedly highlighted the importance of Oct4 in inducing pluripotency. Nanog however was reported to be dispensable in the proposed essential factors in inducing pluripotency.^79^, ^80^, ^81^ It was found that although iPSC induced with the Yamanaka factors did show pluripotent characteristics and show a considerable resemblance to ESC, their epigenetic patterning especially on the *Oct4* and *Nanog* promoter regions showed incomplete demethylation (intermediate between their parental fibroblasts and ESC), and that their embryos did not survive beyond mid‐gestation.^79^, ^84^ Later works demonstrated that the introduction of Nanog alongside the Yamanaka factors led to the formation of iPSC with similar pluripotency characteristics but with very high resemblance to ESC especially with regard to the epigenetic patterning, as well as having more stable ESC marker expression and generation of germline‐competent embryo.^84^, ^85^ It was deduced later that the Yamanaka factors are important in the initial process reprogramming somatic cells to a de‐differentiated state,^84^, ^86^ while Nanog is required to drive the cells to a full ground‐state pluripotency and its maintenance by means of ensuring a more complete epigenetic reprogramming.^86^ Given the importance of Nanog in achieving and maintaining full pluripotency as well as the potential dose dependence in the sensitivity of Nanog action,^86^ the higher *Nanog* expression in DPPSC spheroids when cultured in KO‐medium suggests an improvement in the pluripotency of DPPSC and is therefore desirable. It is noted that the expression of both *Oct4A* and *Nanog* in DPPSCs is lower compared to that of iPSCs (Figure 3), and this is expected since both genes were exogenously introduced during the induction of iPSCs.^79^, ^80^, ^81^ It has been reported that Sox2 is another key factor in induction and maintenance of pluripotency.^87^, ^88^ This has been supported by a study that demonstrated Oct4 and Sox2 as the minimum indispensable factors required to induce pluripotency.^83^ A recent study also reported that polycistronic expression of Klf4 and Sox2 is sufficient to induce pluripotency in the absence of Oct4.^89^ Hence, it will be beneficial in future studies to assess the expression levels of these other key pluripotency‐related genes, among others, in DPPSC spheroids cultured in KO‐medium to further understand how this culture condition influences their pluripotency properties.

It is interesting that the expression of exosomal markers CD63, CD9, Alix and TSG101 are found to be significantly lower on EV_DPPSC_ from day 13‐24 of culture as compared to that from day 1‐12 (Figure 4C‐4F). Since equal numbers of EV_DPPSC_ from each culture period were used for the signal detection by dot blot, this suggests that the EV_DPPSC_ samples from day 13‐24 consisted of a smaller population of exosomes than that from day 1‐12, and that the bulk of the EV population from the former was probably apoptotic bodies. This is supported by the fact that DPPSC spheroids in KO‐medium gradually decreasing in size throughout the culture period, suggesting that they were slowly undergoing apoptosis. Assessing the prevalence of apoptotic markers (eg, Annexin V/PI staining, DNA fragmentation) on the spheroids from day 12 and day 24 would shed some light to this speculation. Annexin V staining could also be done on EV_DPPSC_ samples from both culture periods to identify the population of apoptotic bodies, but this might be inconclusive as phosphatidylserine (PS) were reported to be enriched on exosomal membranes and have been used as a marker to characterize exosomes.^90^, ^91^ Therefore, this implies that CM of DPPSC spheroids in KO‐medium should only be collected up until day 12 of culture to ensure isolation of Exo_DPPSC_ with high purity, thus enabling better correlation with any effects to be studied in downstream experiments. Transplanting DPPSCs into a skin wound in murine models has been reported to promote wound healing by stimulating re‐epithelialization and modulating collagen deposition, through paracrine effects.^16^ Future work will focus on side‐by‐side comparison of the regenerative effects of the DPPSC cells with Exo_DPPSC_ isolated from 3D DPPSC culture.

To conclude, we have demonstrated that the 3D culture of DPPSCs in KO‐medium improved their pluripotency with respect to the augmented *Nanog* expression. This serum‐free culture condition has also enabled the first ever report on the isolation of exosomes from 3D culture of DPPSC (Exo_DPPSC_). We have demonstrated that the 3D culture of DPPSC for 12 days allows for isolation of Exo_DPPSC_ with minimal contamination from nonexosomal vesicles. Future works would include investigating the therapeutic efficacy of Exo_DPPSC_ in vitro and in preclinical animal models. It would also be interesting to investigate the potential of Exo_DPPSC_ in drug delivery applications. It is hoped that the findings of this study would further advance the position and application of DPPSC as a novel source of pluripotent stem cells.

## AUTHORS’ CONTRIBUTIONS

F. N. Faruqu and K. T. Al‐Jamal designed the research; F. Gheidari and H. Lu aided the design and optimization, and provided the tools for qRT‐PCR analysis; F. N. Faruqu, S. Zhou, and N. Sami performed the research; F. N. Faruqu, S. Zhou, and N. Sami analyzed the data; F. N. Faruqu prepared the manuscript; KT Al‐Jamal proofread the manuscript.

## Supporting information

Fig S1

Fig S2

Fig S3

Fig S4

Table S1‐S2
