# Supplementary material for: Three‐dimensional culture of dental pulp pluripotent‐like stem cells (DPPSCs) enhances Nanog expression and provides a serum‐free condition for exosome isolation
Source: FASEB Bioadv. 2020 Jun 28;2(7):419–33. doi: 10.1096/fba.2020-00025 (PMC7354694; doi:10.1096/fba.2020-00025)
Supplement: Supplementary file 1 — Fig S1 [file FBA2-2-419-s001.pdf]

A

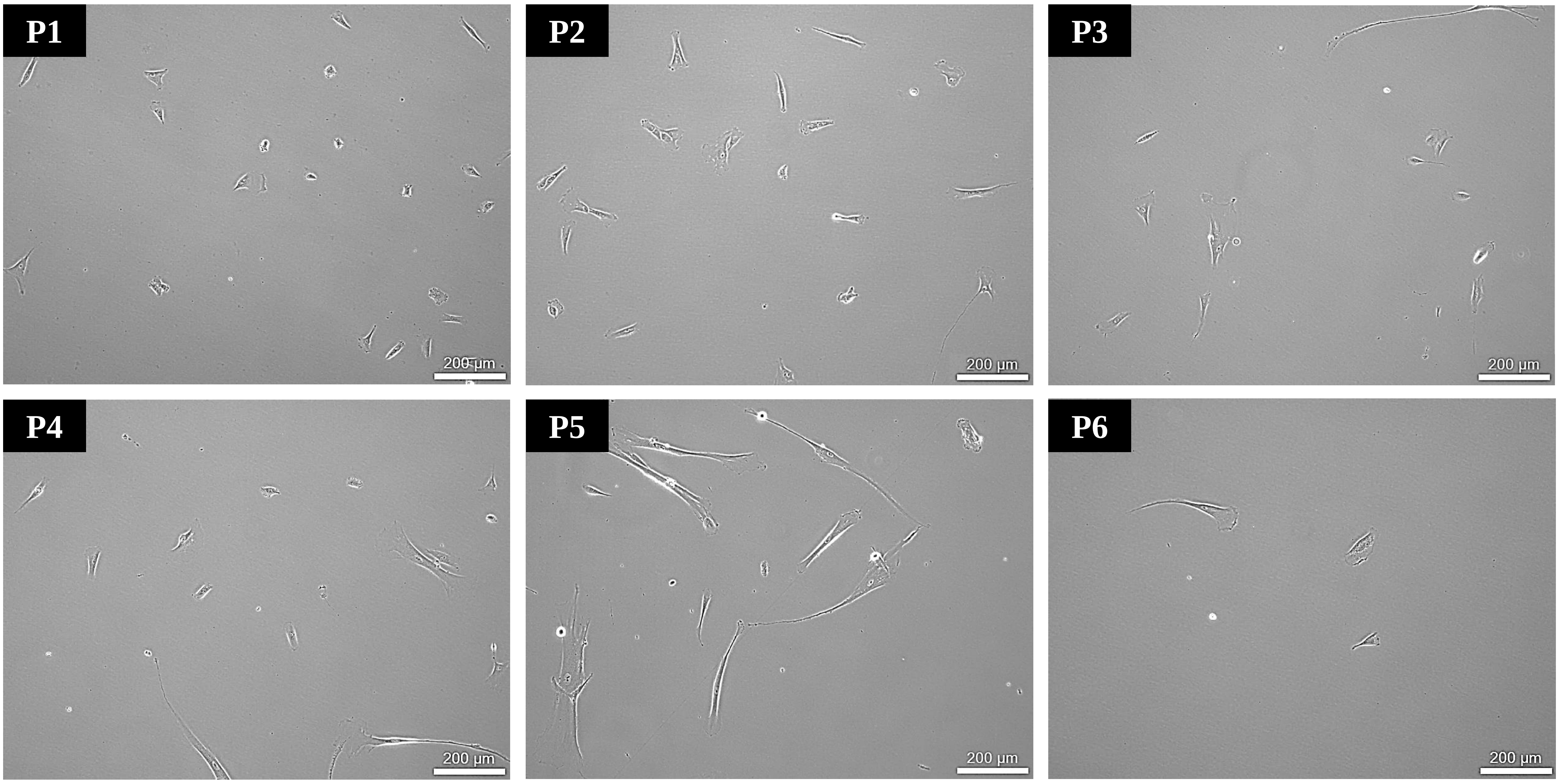


B

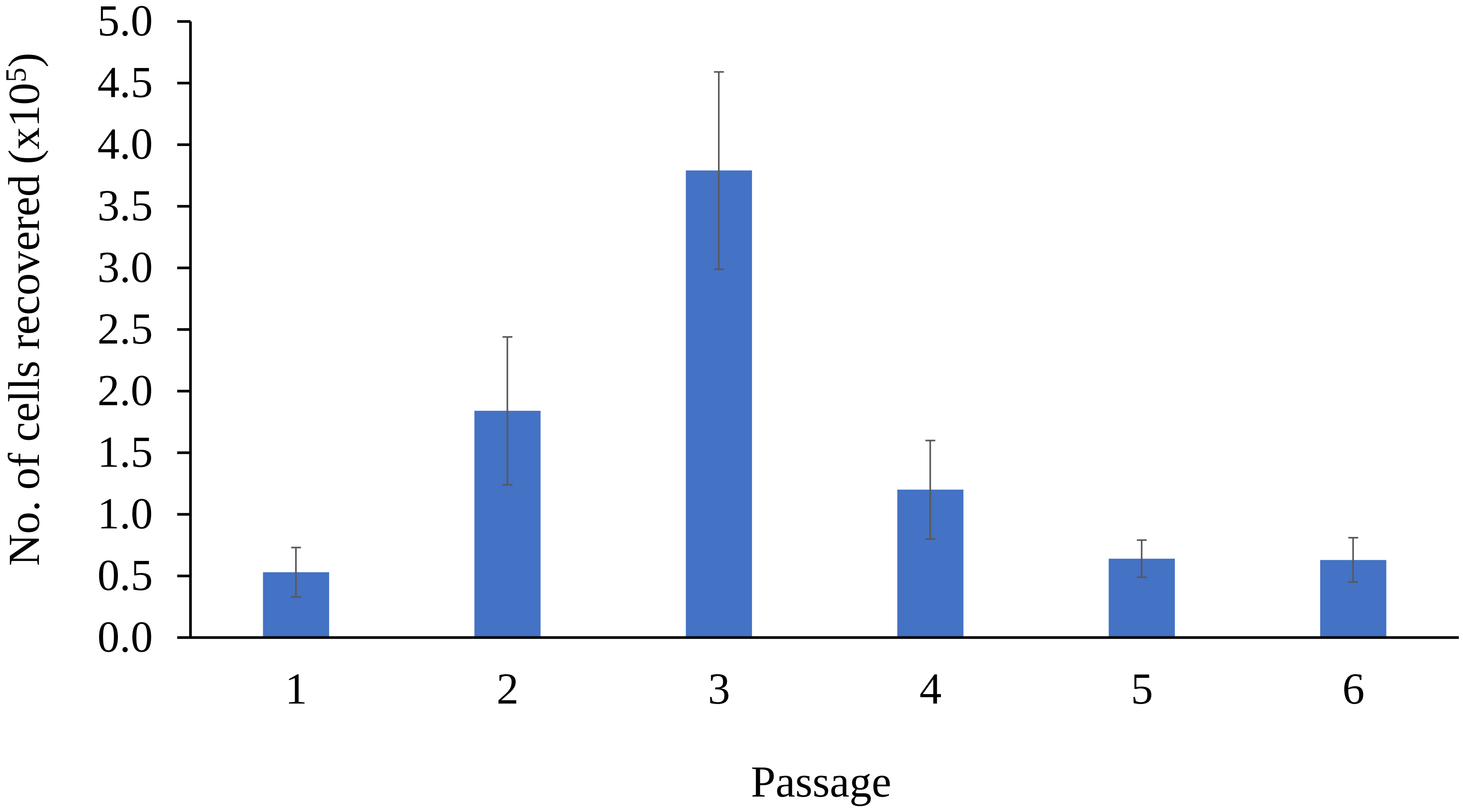


**Fig. S1 2D culture of DPPSC in FBS-supplemented medium. (A)** Representative morphology of DPPSC cultured in FBS-supplemented medium over 6 passages by bright field microscopy under 10X magnification. **(B)** Proliferation of DPPSC in FBS-supplemented medium, assessed by the number of cells recovered from each passage over 5 passages. Values are expressed as mean ± SD, where n=3 different DPPSC clones.
