# Supplementary material for: Three‐dimensional culture of dental pulp pluripotent‐like stem cells (DPPSCs) enhances Nanog expression and provides a serum‐free condition for exosome isolation
Source: FASEB Bioadv. 2020 Jun 28;2(7):419–33. doi: 10.1096/fba.2020-00025 (PMC7354694; doi:10.1096/fba.2020-00025)
Supplement: Supplementary file 2 — Fig S2 [file FBA2-2-419-s002.pdf]

A

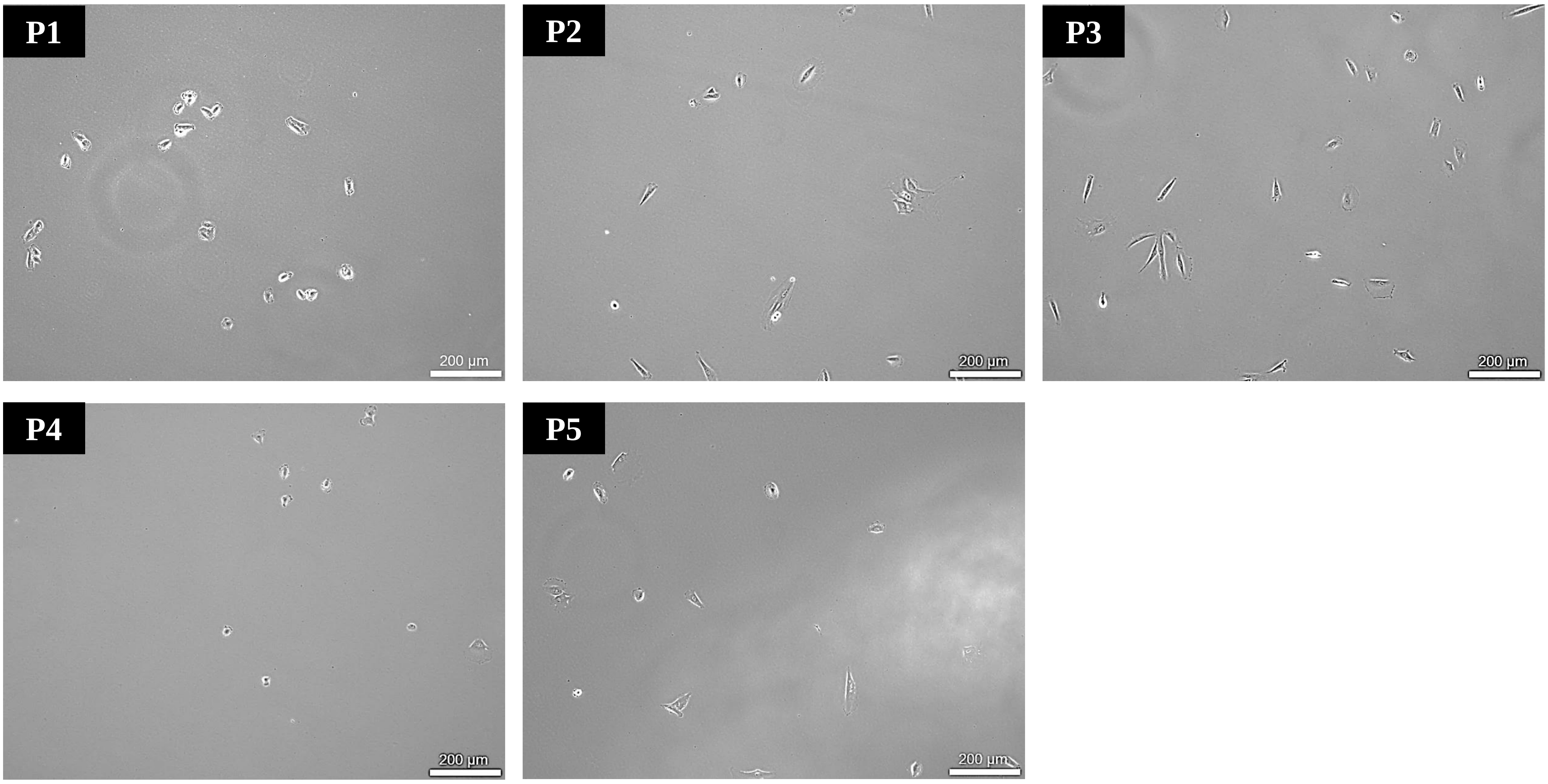

B

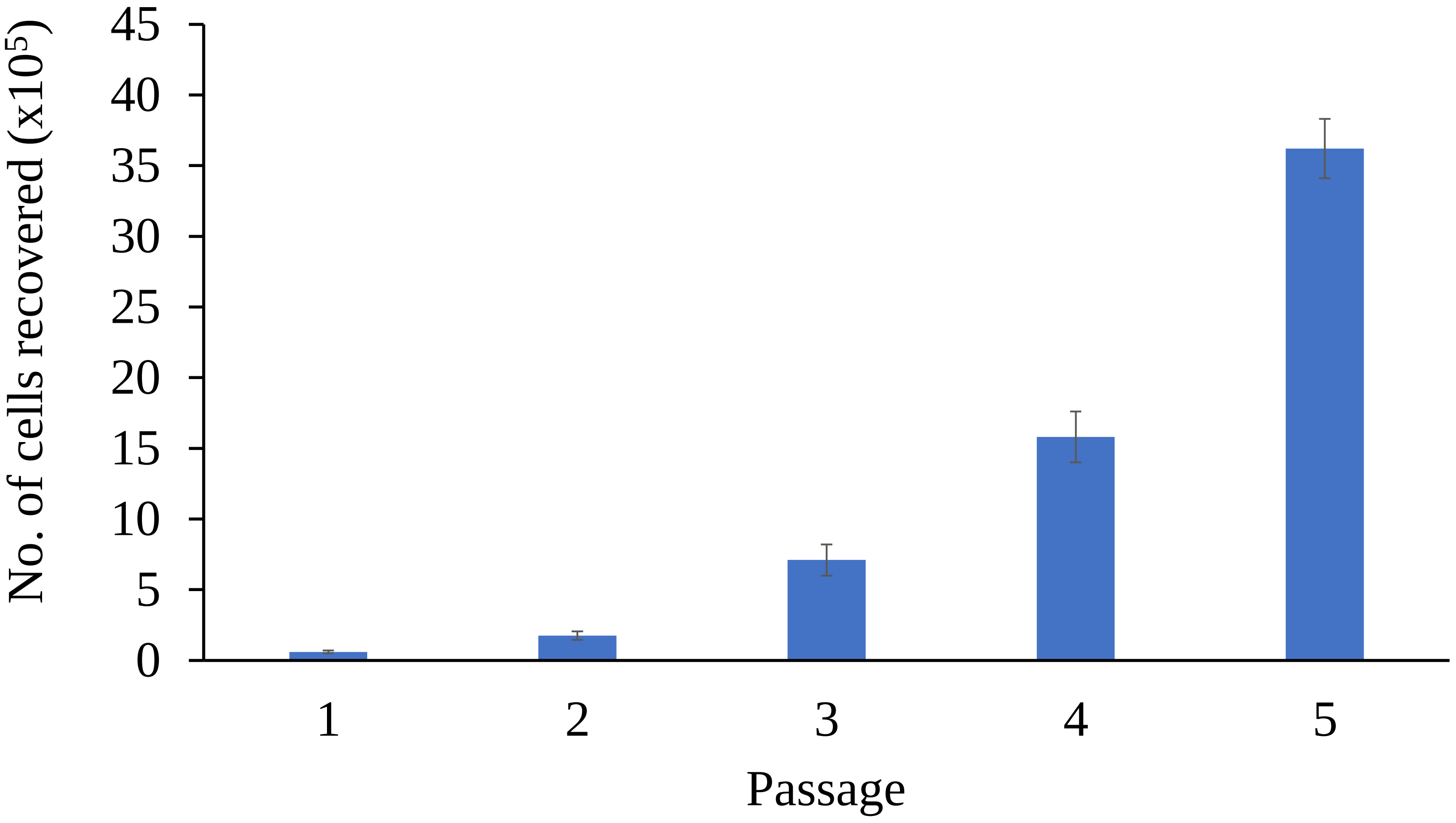

Fig. S2 2D culture of DPPSC in human serum (HS)-supplemented medium. (A) Representative morphology of DPPSC cultured in HS-medium over 5 passages by bright field microscopy under 10X magnification. **(B)** Proliferation of DPPSC in HS-medium, assessed by the number of cells recovered from each passage over 5 passages. Values are expressed as mean ± SD, where n=3 different DPPSC clones.
