# Supplementary material for: Three‐dimensional culture of dental pulp pluripotent‐like stem cells (DPPSCs) enhances Nanog expression and provides a serum‐free condition for exosome isolation
Source: FASEB Bioadv. 2020 Jun 28;2(7):419–33. doi: 10.1096/fba.2020-00025 (PMC7354694; doi:10.1096/fba.2020-00025)
Supplement: Supplementary file 4 — Fig S4 [file FBA2-2-419-s004.pdf]

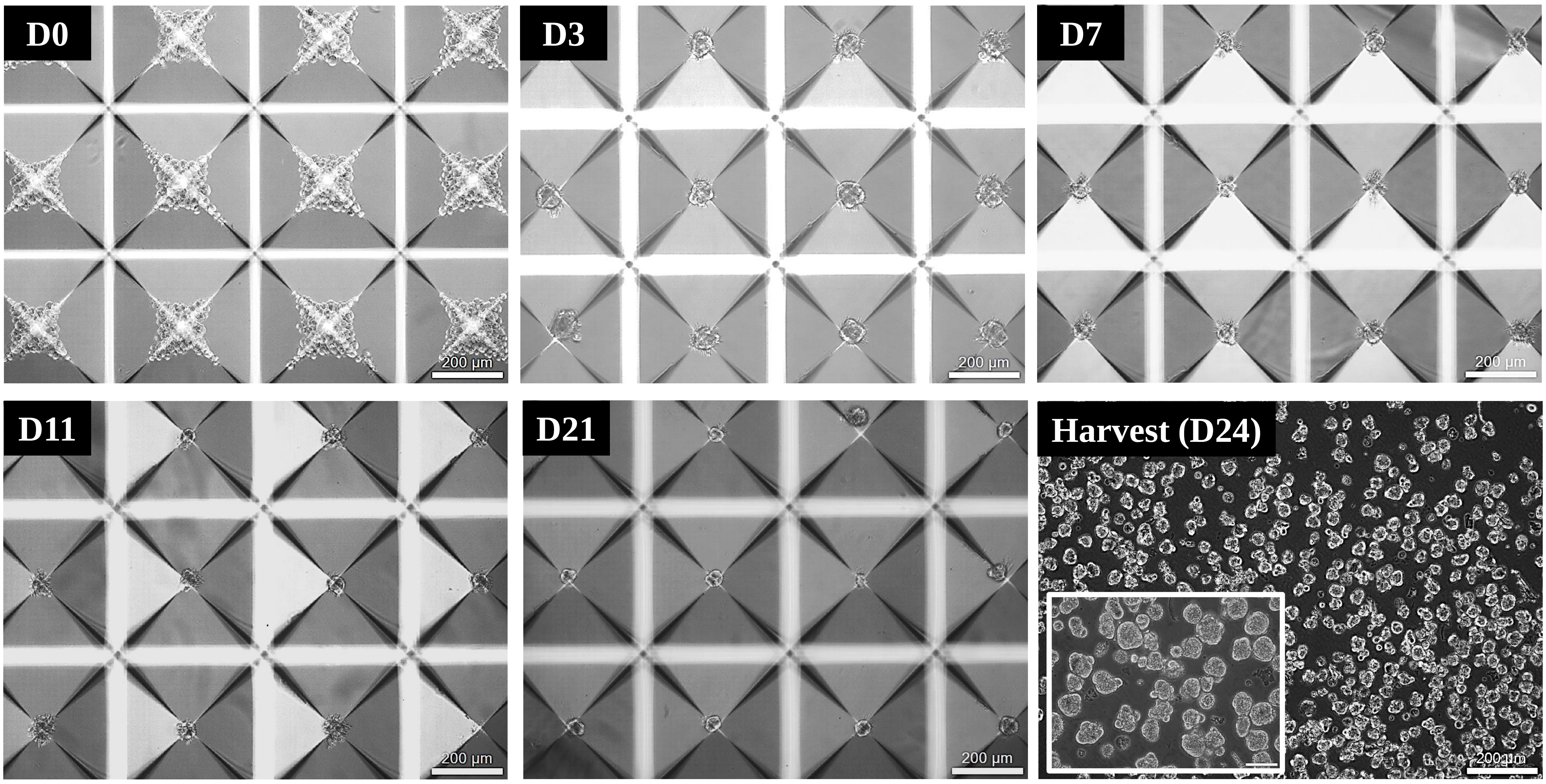

Fig. S4 DPPSC spheroid formation and maintenance in KO-medium in Aggrewell™ plate. Images show representative morphology of DPPSC forming spheroids in KO-medium by bright field microscopy under 10X magnification throughout culture. Inset on image from Day 24 shows zoomed in images of the spheroids post-harvest under 40X magnification. DPPSC were seeded at a density of 1.2 x 10^5 cells/well (100 cells/microwell) of the Aggrewell™ plate.
