# Supplementary material for: Three‐dimensional culture of dental pulp pluripotent‐like stem cells (DPPSCs) enhances Nanog expression and provides a serum‐free condition for exosome isolation
Source: FASEB Bioadv. 2020 Jun 28;2(7):419–33. doi: 10.1096/fba.2020-00025 (PMC7354694; doi:10.1096/fba.2020-00025)
Supplement: Supplementary file 5 — Table S1‐S2 [file FBA2-2-419-s005.docx]

## SUPPLEMENTARY TABLES

1. Proportion of dead cells in culture started to increase
2. Cells did not reach 40% confluency after 7 days and started to predominantly exhibit different morphology. Medium was changed after day 4 of culture
3. From 3 different DPPSC clones

**Table S1 2D culture of DPPSC in FBS-medium**

| **Passage** | **Seeding density cells/cm^2^** | **Days taken to reach**  **40% confluency** | **No. of cells recovered^3^**  **(x10^5^)** |
| --- | --- | --- | --- |
| P1 | 1.65 x 10^5^ total cells  (from thawing) | 2 | 0.53 ± 0.21 |
| P2 | 100 | 4 | 1.84 ± 0.63 |
| P3 | 150 | 3 | 3.79 ± 0.80 |
| P4 | 150 | 4 | 1.20 ± 0.42 |
| P5^1^ | 200 | 4 | 0.64 ± 0.15 |
| P6^2^ | 91 | 7 | 0.63 ± 0.18 |

**Table S2 2D culture of DPPSC in HS-medium**

| **Passage** | **Seeding density cells/cm^2^** | **Days taken to reach**  **40% confluency** |
| --- | --- | --- |
| P1 | 2 x 10^5^ total cells  (from thawing) | 2 |
| P2 | 100 | 4 |
| P3 | 150 | 3 |
| P4 | 100 | 4 |
| P5 | 150 | 3 |
| P6 | 100 | 4 |
